# Curcumin potentiates antitumor activity of cisplatin in bladder cancer cell lines via ROS-mediated activation of ERK1/2

**DOI:** 10.18632/oncotarget.11563

**Published:** 2016-08-24

**Authors:** Bong Hee Park, Joung Eun Lim, Hwang Gyun Jeon, Seong Il Seo, Hyun Moo Lee, Han Yong Choi, Seong Soo Jeon, Byong Chang Jeong

**Affiliations:** ^1^ Department of Urology, Samsung Medical Center, Sungkyunkwan University School of Medicine, Seoul, Republic of Korea; ^2^ Samsung Biomedical Research Institute, Samsung Medical Center, Seoul, Republic of Korea; ^3^ Department of Urology, Uijeongbu St. Mary's Hospital, College of Medicine, The Catholic University of Korea, Seoul, Republic of Korea

**Keywords:** bladder cancer, cisplatin, curcumin, ERK, oxidative stress

## Abstract

Resistance of bladder cancer to cisplatin is a major obstacle to successful treatment. In the current study, we investigated the apoptotic effects of curcumin and cisplatin co-treatment in 253J-Bv(p53 wild-type) and T24(p53 mutant) bladder cancer. We found that curcumin and cisplatin co-treatment primarily targets reactive oxygen species(ROS) and extracellular regulated kinase(ERK) signaling during the apoptosis induction in bladder cancer. The apoptosis rate in 253J-Bv and T24 cells co-treated with curcumin and cisplatin was increased compared to that in cells exposed to single-agent treatment conditions. Also, caspase-3 activation and ROS production were observed in both cells treated with curcumin and cisplatin, together with upregulation of p-MEK and p-ERK1/2 signaling. NAC(ROS scavenger) and U0126(ERK inhibitor) inhibited apoptosis induced by curcumin and cisplatin. In addition, when 253J-Bv cells were co-treated with curcumin and cisplatin, p53 and p21 expression levels were markedly increased when compared to controls. Unlike 253J-Bv cells, T24 cells were co-treated with curcumin and cisplatin revealed an induction of apoptosis through decreased p-signal transducer and activator of transcription 3(STAT3) expression. Moreover, pretreatment with U0126 suppressed curcumin and cisplatin-induced upregulation of p53, p21, and p-STAT3 and downregulation of survival proteins in both cells. In conclusion, co-treatment with curcumin and cisplatin synergistically induced apoptosis through ROS-mediated activation of ERK1/2 in bladder cancer.

## INTRODUCTION

Bladder cancer is one of the most common genitourinary malignant diseases and represents a serious health problem worldwide [1]. At diagnosis, approximately 70% of cancers are non-muscle invasive tumors and the remaining 30% are muscle invasive [2]. However, among non-muscle invasive tumors, as many as 50-70% recur and approximately 10-20% progress to muscle invasive bladder cancer [3]. In addition, about 10% of patients diagnosed with muscle invasive tumors have metastatic disease with a poor prognosis [2].

Cisplatin-based combination chemotherapy has evolved as standard therapy for advanced or metastatic bladder cancer [4], showing a 50-70% response rate and 15-20% improved survival. However, approximately 30% of patients do not respond to initial chemotherapy and most have recurrence within 1 year [5]. Cisplatin is an inorganic platinum agent that can bind to DNA, inducing intrastrand and interstrand DNA crosslinks as well as DNA-protein crosslinks [6]. Although these crosslinks result in apoptosis and cell growth inhibition [7], cisplatin efficacy is commonly decreased by the development of cell resistance. Therefore, there are needs to develop novel treatment strategies that have fewer side effects and are more effective than the currently used therapeutic regimens for bladder cancer.

Curcumin is a natural polyphenol compound derived from turmeric, the powdered rhizome of the medicinal plant *Curcuma longa Linn* [8]. *In vitro* and *in vivo* preclinical studies have shown that curcumin has antioxidant, anti-inflammatory, antiproliferative, and proapoptotic activities [9]. Recent studies have shown that curcumin could be an effective chemopreventive and chemotherapeutic agent in bladder cancer [10]. Curcumin targets diverse molecules associated with numerous biochemical and molecular cascades via direct molecular interactions and/or epigenetic modulation of gene expression [11]. However, the molecular basis for the curcumin effects is not fully understood.

Several studies indicate that curcumin possesses ROS-inducing or pro-oxidant activity [12, 13]. Since cellular oxidative stress induced by cisplatin has been shown to contribute to its cytotoxic activity and increased antioxidant mechanisms of cancer cells attenuate cisplatin-induced apoptosis [14, 15], the pro-oxidant property of curcumin may increase the cisplatin efficacy for cancer management. Various animal models and human studies proved that curcumin is non-toxic even at high doses [16, 17]. Therefore, curcumin is a remarkable candidate for the therapeutic strategies development for cancer management.

We examined whether curcumin synergistically potentiated the anticancer activity of cisplatin in two different human bladder cancer cell lines. We additionally evaluated the possible molecular signaling pathway underlying this effectiveness.

## RESULTS

### Curcumin potentiates the antiproliferative efficacy of cisplatin in human bladder cancer cell lines

**Figure 1:**
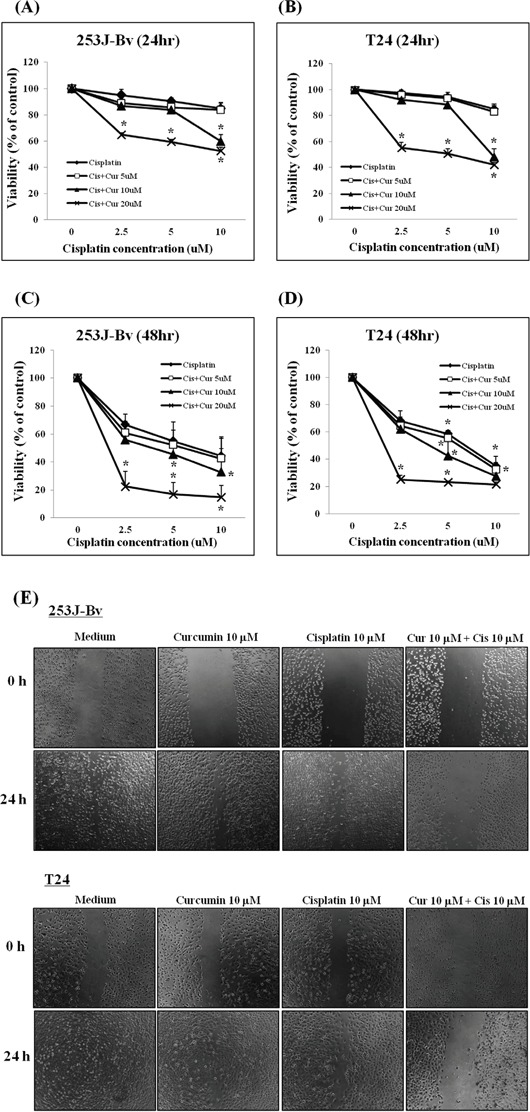
Proliferation rates of 253J-Bv and T24 cells after treatment with various cisplatin or curcumin concentrations **A-D.** Human bladder cancer cell lines (253J-Bv and T24) were treated with curcumin (5, 10, or 20 μM) and cisplatin (2.5, 5, or 10 μM) for 24 and 48 h. Cancer cell viability was measured by MTT assay. Data are expressed as the mean ± SEM of three independent experiments. *p <0.005 compared with medium alone was considered statistically significant. **E.** 253J-Bv and T24 cell monolayers were carefully scratched with a pipette tip and subsequently incubated with cisplatin (10 μM) and curcumin (10 μM) for 24 h. No treatment was administered to the control cancer cells. Migrating cells were photographed at 0 and 24 h post-wounding under a phase contrast microscope. The images represent three experiments showing similar results.

The cytotoxic efficacy of co-treatment with curcumin and cisplatin was determined in human bladder cancer 253J-Bv and T24. Bladder cancer cells were incubated with 2.5–10 μM cisplatin alone or in combination with 5-20 μM curcumin for 24 and 48 h, and cancer cell viability was investigated by MTT assay. Figure 1A-1D shows that treatment with cisplatin and curcumin reduced the viability of 253J-Bv and T24 cells in a time- and dose-dependent fashion compared with medium alone. Co-treatment with cisplatin and curcumin exhibited significant cytotoxicity at 10 μM for each drug (Figure 1A and 1B). Cancer cell migration inhibition was assessed by a wound-healing assay with 253J-Bv and T24 cells. Cells in medium displayed a higher migration rate to the scratched wound area relative to drug-treated cells. Moderate inhibition of migration was detected in cancer cells treated with either curcumin or cisplatin, whereas a significant inhibition of migration was observed for cells co-treated with curcumin and cisplatin (Figure 1E).

### Curcumin potentiates apoptotic effects induced by cisplatin in 253J-Bv and T24 cells

**Figure 2:**
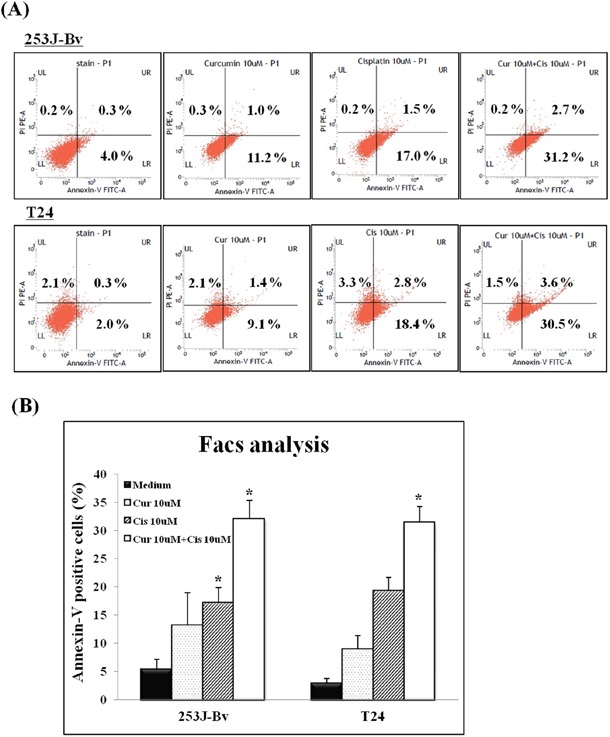
Detection of apoptotic cells in 253J-Bv and T24 bladder cancer cell cultures using flow cytometry after annexin V-FITC/PI staining 253J-Bv and T24 cells were incubated for 24 h at 37°C with or without 10 μM curcumin and 10 μM cisplatin. After incubation, cancer cells were stained with PI and FITC-conjugated annexin V for flow cytometry. The diagram shows binding of annexin V (FL1) and PI in bladder cancer cells incubated for 24h with or without curcumin and cisplatin. The percentage of bladder cancer cells stained with both PI and FITC-labeled annexin V in each sample is indicated. Data are expressed as the mean ± SEM of three independent experiments. Significant differences from the results obtained for cells incubated with only medium are shown. *p <0.005 compared with medium alone was considered statistically significant.

We further evaluated whether combination treatment increases apoptotic events in cancer cells. 253J-Bv and T24 cells were treated with or without curcumin (10 μM) and cisplatin (10 μM) for 24 h followed by annexinV-FITC/PI staining for flow cytometry. As shown in Figure 2A, curcumin or cisplatin alone induced 253J-Bv and T24 cells apoptosis following 24 h drug exposure and this impact was enhanced when the agents were concurrently treated as a combination therapy. The apoptotic percentage of the untreated 253J-Bv cells was 4.3%, which increased to 18.5% and 12.2% after treatment with cisplatin or curcumin, respectively. Following co-treatment with curcumin and cisplatin, the apoptotic percentage significantly increased to 33.9% (Figure 2B). For T24 cells, the apoptotic percentage in the untreated cells was 2.3%, which increased to 21.2% and 10.5% following treatment with cisplatin or curcumin and significantly increased to 34.1% following co-treatment with cisplatin and curcumin (Figure 2B).

### Curcumin and cisplatin induce caspase 3-mediated apoptosis in bladder cancer cell lines

**Figure 3:**
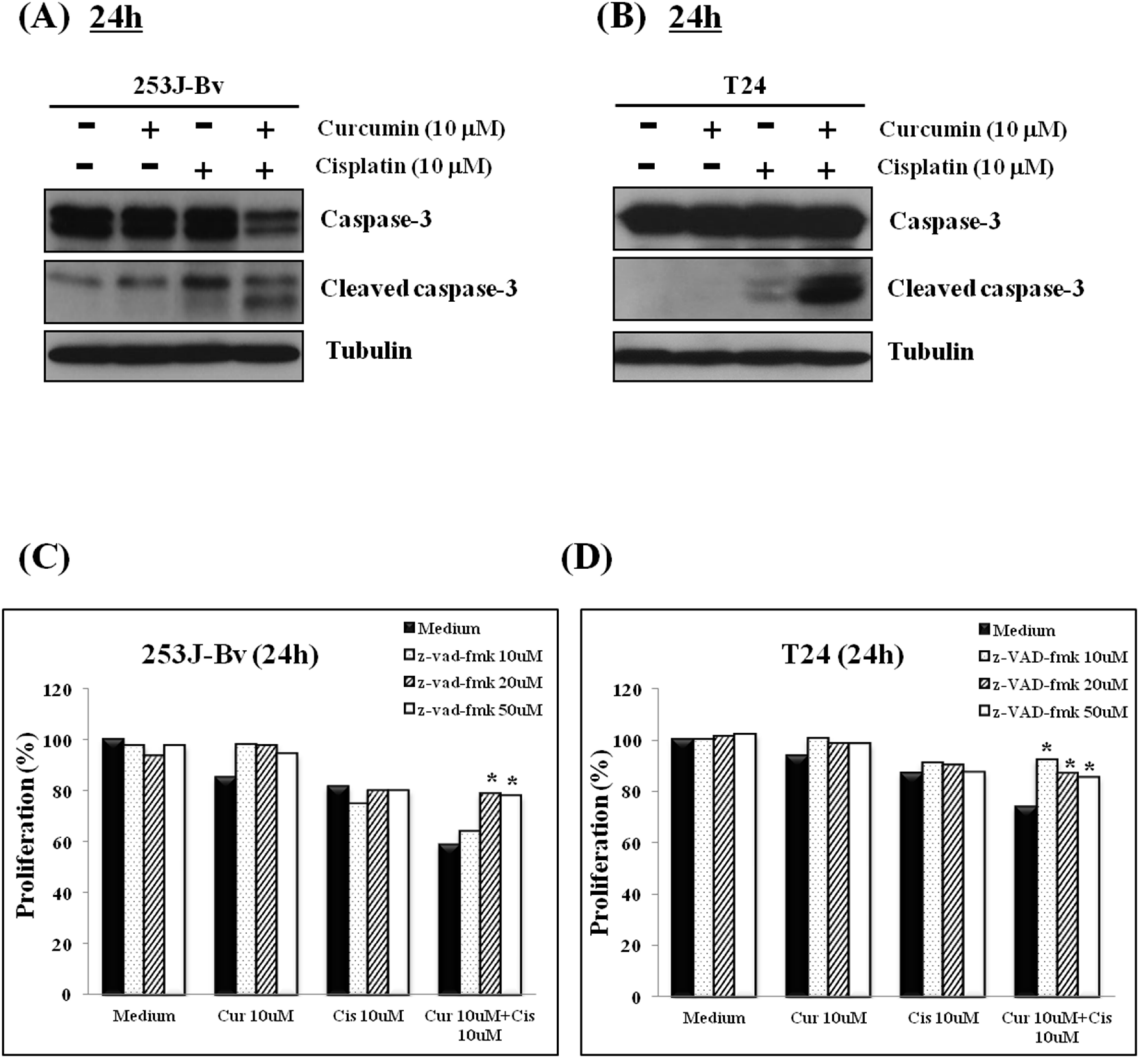
Involvement of caspase 3 in apoptosis of 253J-Bv and T24 bladder cancer cells incubated with curcumin and cisplatin **A, B.** 253J-Bv and T24 cells were incubated for 24 h with or without 10 μM curcumin and 10 μM cisplatin. After incubation, samples were subjected to SDS-PAGE and immunoblotted with anti-caspase 3 or anti-tubulin antibody. The figure represents three experiments showing similar results. **C, D.** 253J-Bv and T24 cells pretreated with Z-VAD-FMK (10, 20, and 50 μM) for 1 h were incubated with or without 10 μM curcumin and 10 μM cisplatin for 24 h. After incubation, cancer cell viability was measured by MTT assay. Data are expressed as the mean ± SEM of three independent experiments. Significant differences from the results obtained for cells incubated with only medium are shown. *p <0.005 compared with medium alone was considered statistically significant.

Curcumin and cisplatin have both been reported to induce apoptosis through intracellular caspase 3 activation. To assess whether caspase 3 activation was involved in apoptosis of bladder cancer cells induced by the combination treatment, 253J-Bv and T24 cells were treated with or without 10 μM curcumin and 10 μM cisplatin for 24 h. As shown in Figure 3A and 3B, increased the cleaved form levels of caspase 3 were detected in the lysates of cells applied with cisplatin and curcumin co-treatment compared with cells incubated with curcumin or cisplatin alone. Moreover, the induction of cell proliferation in bladder cancer by curcumin and cisplatin co-treatment was significantly inhibited by pretreatment of the cells with the pan-caspase inhibitor, Z-VAD-FMK (Figure 3C and 3D). These outcomes suggest evidence that caspase 3 is involved in apoptosis mediated by combination curcumin and cisplatin treatment in 253J-Bv and T24 cells.

### Curcumin and cisplatin promote reactive oxygen species (ROS) generation in 253J-Bv and T24 cells

**Figure 4:**
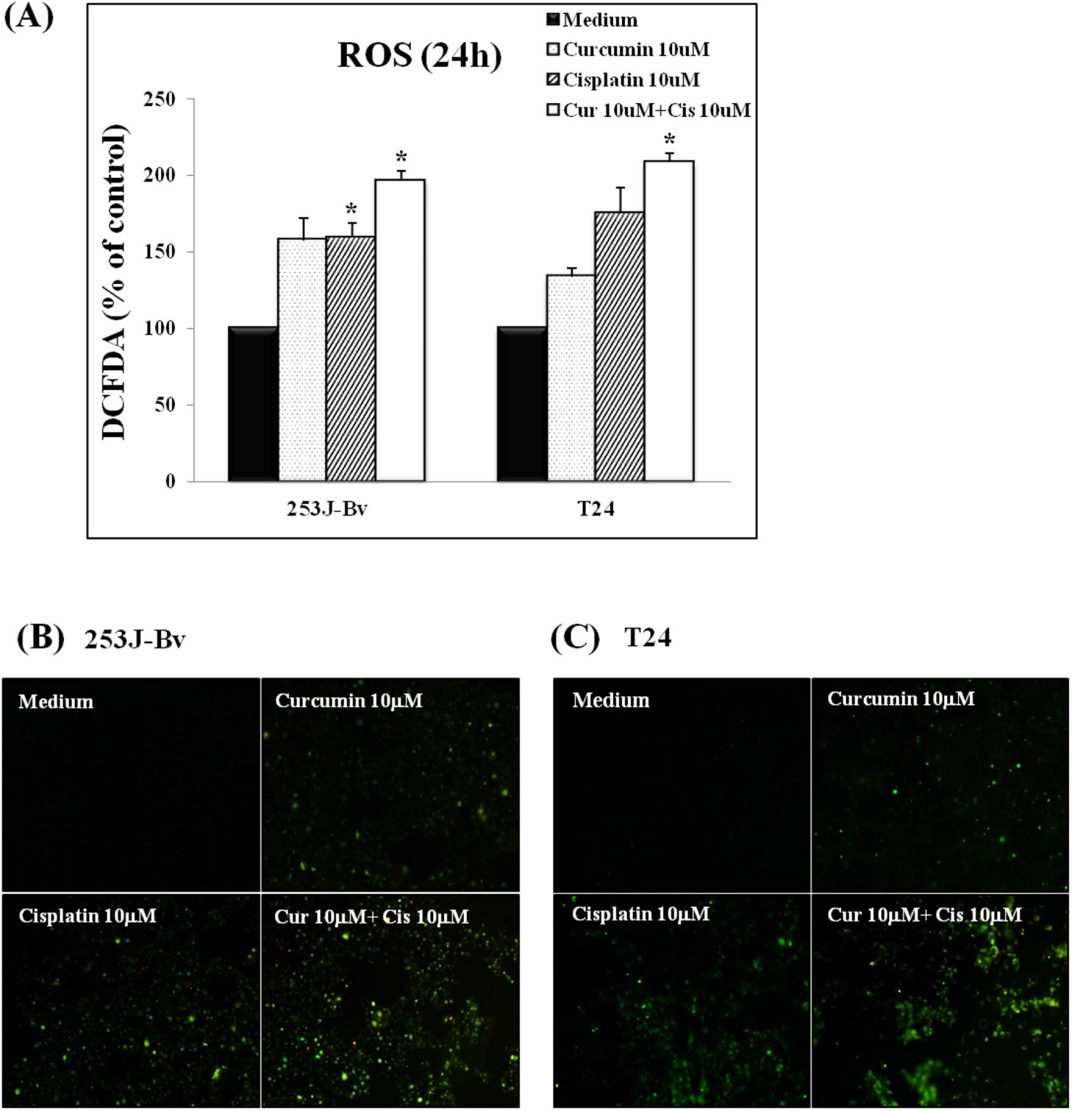
ROS generation induced by curcumin in combination with cisplatin in 253J-Bv and T24 bladder cancer cells **A.** 253J-Bv and T24 cells were incubated with 10 μM cisplatin and 10 μM curcumin for 24 h and then stained with 10 μM H2DCFDA to assess intracellular ROS levels by flow cytometry. Data are normalized to the mean fluorescence intensity of DCF in 253J-Bv and T24 cells stimulated without curcumin and cisplatin, taken as 100%. Data are expressed as the mean ± SEM of three independent experiments. Significant differences from the results obtained with cells incubated with only medium are shown. *p <0.005 compared with medium alone was considered statistically significant. **B, C.** 253J-Bv and T24 cells were incubated with 10 μM cisplatin and 10 μM curcumin for 24 h. The intracellular ROS generation in bladder cancer cells was detected by inverted fluorescence microscopy (400× magnification). The images represent three experiments showing similar results.

ROS plays important roles in apoptosis of cancer cells in response to DNA damage. Therefore, to investigate whether co-treatment with curcumin and cisplatin induced intracellular ROS generation in 253J-Bv and T24 cells, the cells were stained with H2DCFDA. When 253J-Bv and T24 cells were co-incubated with cisplatin and curcumin for 24 h, the average fluorescence intensity of DCF as detected by flow cytometry was approximately 2-fold greater than that of bladder cancer cells incubated with only medium (Figure 4A). In addition, co-treatment with curcumin and cisplatin clearly induced intracellular green ROS fluorescence in 253J-Bv and T24 cells as detected by fluorescent microscopy. As shown in Figure 4B and 4C, 253J-Bv and T24 bladder cells co-incubated with curcumin and cisplatin showed intense ROS response (as indicated by green fluorescence) by fluorescence microscopy compared to bladder cancer cells incubated with only medium. These observations indicate that cell apoptosis induced by co-treatment with curcumin and cisplatin is associated with ROS production in 253J-Bv and T24 cells.

### Curcumin and cisplatin upregulate p-ERK1/2 signaling in 253J-Bv and T24 cells

**Figure 5:**
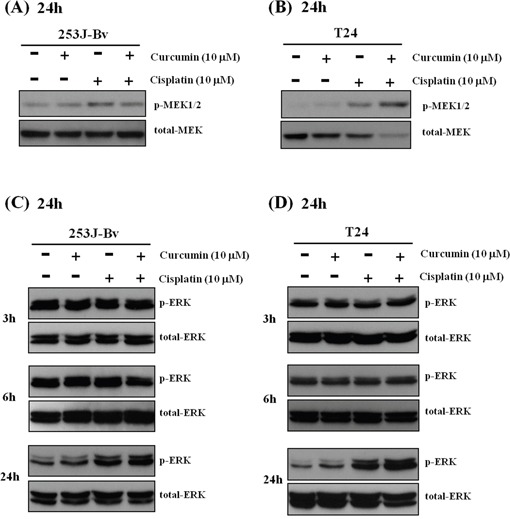
Activation of p-MEK and p-ERK1/2 in 253J-Bv and T24 bladder cancer cells stimulated with curcumin and cisplatin **A, B.** 253J-Bv and T24 cells were incubated for 24 h at 37°C with or without 10 μM curcumin and 10 μM cisplatin. After incubation, samples were subjected to SDS-PAGE and immunoblotted with anti-phospho-MEK or anti-MEK antibodies. The images represent three experiments showing similar results. **C, D.** 253J-Bv and T24 cells were incubated for 3-24 h with or without 10 μM curcumin and 10 μM cisplatin and immunoblotted with anti-phospho-ERK1/2 or anti-ERK1/2 antibodies. The images represent three experiments showing similar results.

The ERK signaling pathways play a critical role in cells growth and proliferation; however, ERK overactivation may initiate apoptotic cell death by activating intrinsic and extrinsic pathways. We examined the activation status of p-MEK and p-ERK1/2 in 253J-Bv and T24 cells incubated with curcumin and cisplatin. As shown in Figure 5A and 5B, curcumin or cisplatin alone increased the expression of p-MEK, and the two drugs together markedly increased p-MEK expression in 253J-Bv and T24 cells. Curcumin or cisplatin alone also increased the expression of p-ERK, and the protein levels of p-ERK1/2 were strikingly upregulated in 253J-Bv and T24 cells after co-treatment with curcumin and cisplatin for 24 h (Figure 5C and 5D). These results indicate that an increase in p-ERK and p-MEK is another crucial event in the response to combined drug treatment and that curcumin promotes the cisplatin-induced phosphorylation of ERK.

### ROS and ERK are correlated with apoptosis induction in curcumin and cisplatin co-treated 253J-Bv and T24 cells

**Figure 6:**
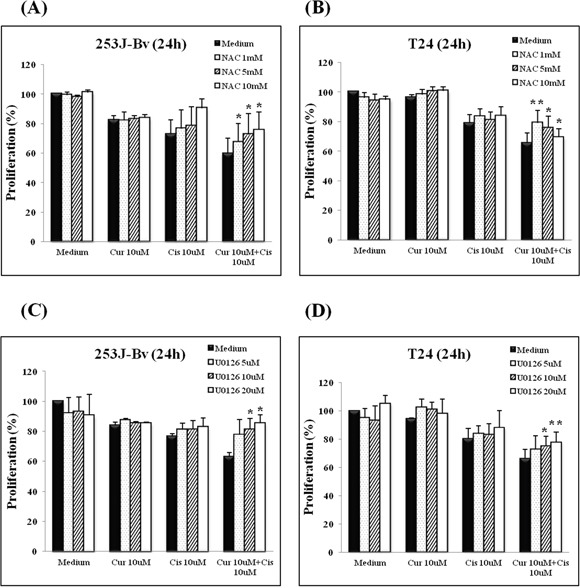
Effect of ROS scavenger (NAC) or MEK inhibitor (U0126) on proliferation of 253J-Bv and T24 bladder cancer cells incubated with curcumin and cisplatin **A-D.** 253J-Bv and T24 cells were pretreated with NAC (1, 5, and 10 mM) or U0126 (5, 10 and 20 μM) for 1 h at 37°C before incubation for 24 h at 37°C with or without 10 μM curcumin and 10 μM cisplatin. After incubation, cancer cell viability was measured by MTT assay. Data are expressed as the mean ± SEM of three independent experiments. Significant differences from the results obtained with cells incubated with only medium are shown. *p <0.05 and **p <0.005 compared with medium alone were considered statistically significant. No cytotoxicity of NAC or U0126 at the treated concentrations was observed.

To assess the roles of ROS and ERK in curcumin and cisplatin-induced apoptosis, we pretreated cells with NAC (ROS scavenger) and U0126 (ERK inhibitor). As shown in Figure 6A and 6B, pretreatment with NAC effectively inhibited curcumin and cisplatin-induced cell death in 253J-Bv and T24 cells. Also, the data in Figure 6C and 6D show that, as with NAC, U0126 reversed the cell viability reduction induced by the combination treatment in 253J-Bv and T24 cells. Collectively, these results suggest that curcumin-provoked ROS generation and ERK phosphorylation may explain the potentiation of cisplatin-induced apoptosis by curcumin.

### Curcumin and cisplatin treatment markedly increases expression of p53 and p21 in 253J-Bv cells and decreases p-STAT3 protein levels in T24 cells

**Figure 7:**
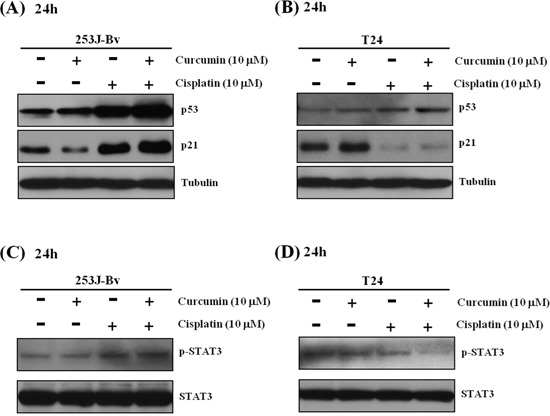
Effect of curcumin combined with cisplatin on p53, p21, and p-STAT3 expression in 253J-Bv and T24 bladder cancer cells **A-D.** 253J-Bv and T24 cells were incubated for 24 h with or without curcumin and cisplatin. After incubation, samples were subjected to SDS-PAGE gels and immunoblotted with antibodies against p53, p21, p-STAT3, and STAT3. The images represent three experiments showing similar results.

We next investigated the efficacy of curcumin and cisplatin co-treatment on cell-cycle regulatory proteins, including p53 and p21, in 253J-Bv and T24 cells (Figure 7A and 7B). In p53 wild-type 253J-Bv cells, curcumin and cisplatin combination treatment resulted in a significant increase in p53 and p21 levels compared with curcumin or cisplatin alone. However, p53-mutant T24 cells showed a partial increase in p53 expression, but no p21 expression, when exposed to curcumin and cisplatin combination treatment. We further analyzed p-STAT3 protein levels in both cell lines after treatment with combination therapy. In T24 cells, curcumin and cisplatin combination treatment markedly inhibited p-STAT3 expression compared to curcumin or cisplatin alone (Figure 7D), and this downregulation was not observed in 253J-Bv cells (Figure 7C). These data suggest that combination treatment might in part target p53 and p21 proteins in 253J-Bv cells and p-STAT3 in T24 cells.

### Curcumin and cisplatin co-treatment reduces apoptosis-regulatory proteins in 253J-Bv and T24 cells

**Figure 8:**
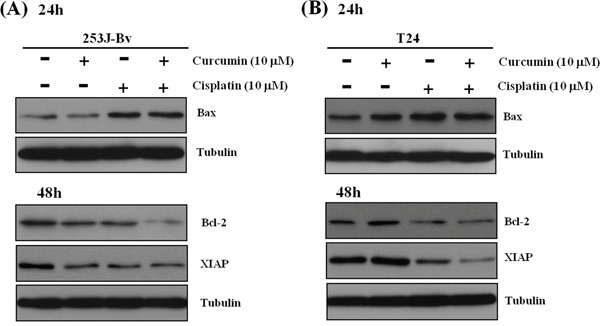
Effects of curcumin combined with cisplatin on the expression of apoptosis-related proteins in 253J-Bv and T24 bladder cancer cells **A, B.** 253J-Bv and T24 cells were incubated for 24 and 48 h with or without 10 μM curcumin and 10 μM cisplatin. After incubation, samples were subjected to SDS-PAGE gels and immunoblotted with antibodies against Bax, Bcl-2, and XIAP. The images represent three experiments showing similar results.

We examined the impact of curcumin and cisplatin on the proapoptotic and antiapoptotic proteins expression by Western blotting. As shown in Figure 8A and 8B, curcumin or cisplatin alone had minimal effects on Bcl-2, Bax, and XIAP expression levels in 253J-Bv and T24 cells. However, the co-treatment resulted in a substantial decline in Bcl-2 and XIAP expression, and an increase in Bax expression, in both cell lines.

### Effects of U0126 on p-ERK1/2 activation, downregulation of p-STAT3, and apoptosis-regulatory proteins induced by combination treatment

**Figure 9:**
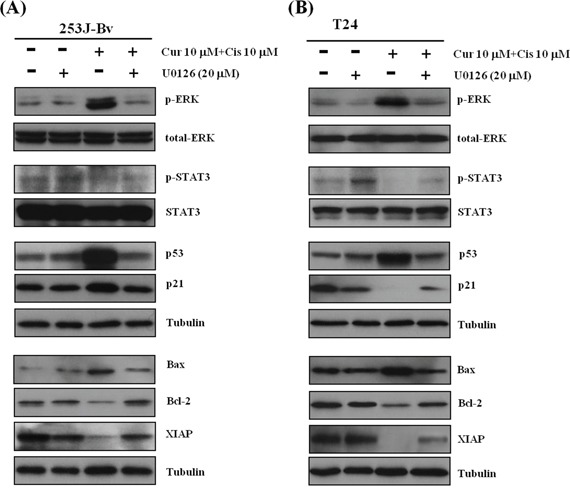
Effect of the MEK inhibitor U0126 on the expression of p-ERK1/2, STAT3 and apoptotic proteins of 253J-Bv and T24 cells treated with curcumin and cisplatin **A, B.** 253J-Bv and T24 cells were pretreated with 20 μM U0126 and then incubated for 24 h or 48 h with or without 10 μM curcumin and 10 μM cisplatin. After incubation, samples were subjected to SDS-PAGE gels and immunoblotted with antibodies against phospho-ERK1/2, ERK1/2, p-STAT3, STAT3, p53, p21, Bax, Bcl-2, or XIAP. The images represent three experiments showing similar results.

We assessed the role of ERK in downregulation of p-STAT3 and apoptosis-regulatory proteins during curcumin and cisplatin-mediated apoptosis. In 253J-Bv cells co-treated with curcumin and cisplatin, U0126 treatment significantly decreased p53 and p21 levels, substantially decreased Bax expression, and substantially increased Bcl-2 and XIAP expression (Figure 9A). However, unlike T24 cells, there was no change in p-STAT3 expression. In T24 cell with cisplatin and curcumin co-treatment, U0126 resulted in a significant increase in p-STAT3, a substantial decrease in the expression of Bax, and a substantial increase in Bcl-2 and XIAP expression (Figure 9B). These results revealed that ERK acts upstream of STAT-3 and p53 in the signaling pathway that leads to apoptosis after curcumin and cisplatin combination treatment.

### Effects of NAC on p-ERK1/2 activation and downregulation of apoptosis-regulatory proteins induced by combination treatment

**Figure 10:**
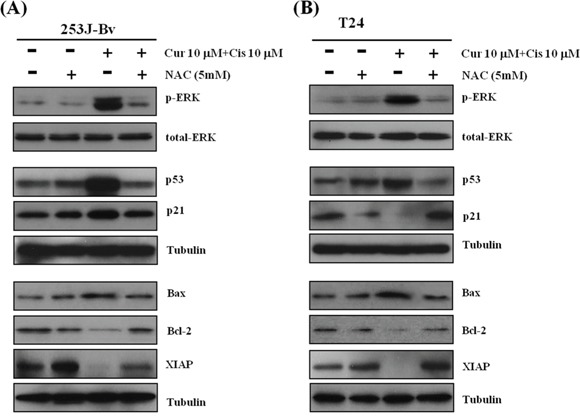
Effect of the ROS scavenger NAC on the expression of p-ERK1/2, STAT3, and apoptotic proteins in 253J-Bv and T24 cells treated with curcumin and cisplatin **A, B**. 253J-Bv and T24 cells were initially treated with 5 mM NAC and then incubated for 24 h or 48 h with or without 10 μM curcumin and 10 μM cisplatin. After incubation, samples were subjected to SDS-PAGE gels and immunoblotted with antibodies against phospho-ERK1/2, ERK1/2, p-STAT3, STAT3, p53, p21, Bax, Bcl-2, or XIAP. The images represent three experiments showing similar results.

To investigate ROS involvement in combination treatment-induced p-ERK1/2 activation, we pretreated 253J-Bv and T24 cells with NAC before incubation with curcumin and cisplatin. As shown in Figure 10A and 10B, NAC inhibited p-ERK1/2 activation after co-treatment with cisplatin and curcumin in both cell lines. Consistent with the effects of U0126, pretreatment with NAC effectively inhibited the increase in p53 and p21 protein levels in 253J-Bv cells, but not in T24 cells (Figure 10A and 10B). Furthermore, NAC also blocked the downregulation of apoptosis-regulatory proteins induced by the combination treatment. These findings indicate that ROS function upstream of curcumin and cisplatin combination-induced p-ERK1/2 activation.

### Curcumin potentiates the antitumorigenic activities of cisplatin in human bladder cancer cells in nude mice bearing 253J-Bv cells

**Figure 11:**
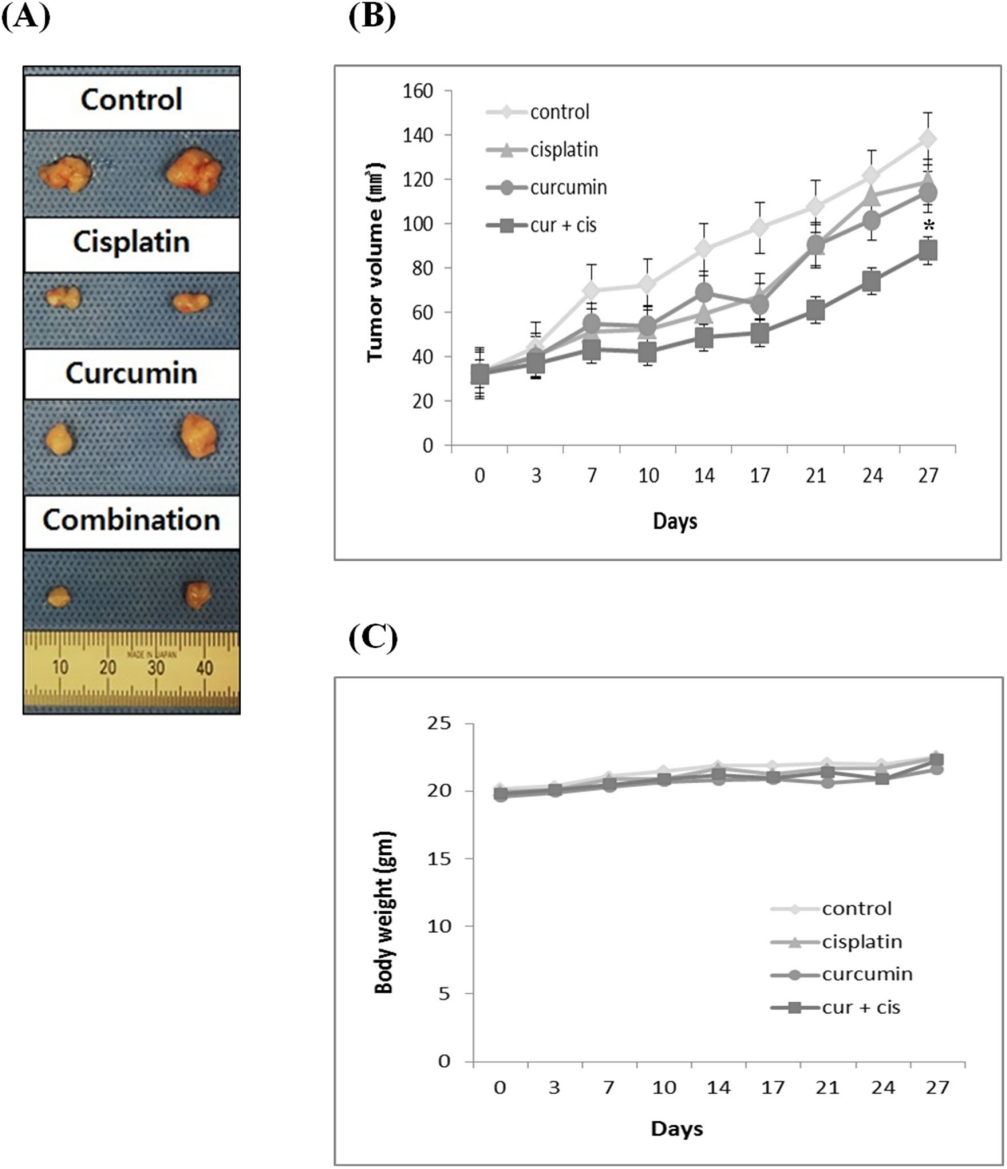
Curcumin potentiates the antitumor activity of cisplatin against human bladder cancer cells in nude mice Animals bearing 253J-Bv cell xenografts were treated with curcumin (500 mg/kg) or cisplatin (5 mg/kg) alone or in combination. Animals were classified into four groups (n=10): the control group was treated with saline; group 1 was treated with cisplatin alone (5 mg/kg, i.p./weekly); group 2 was treated with curcumin alone (500 mg/kg, orally/thrice weekly); and group 3 was treated with the combination of curcumin (500 mg/kg, orally/thrice weekly) and cisplatin (5 mg/kg, i.p./weekly). **A.** Representative photomicrographs of 253J-Bv tumors from control, cisplatin, curcumin, or combination treated groups on the last day of the experiment. **B, C.** Tumor volumes and body weights were measured at indicated time intervals. Point, mean; bar, SEM. *p <0.05 compared with results of the control group was considered statistically significant.

Finally, we investigated the effects of curcumin and cisplatin alone and in co-treatment in nude mice bearing 253J-Bv cell xenografts. Animals of all groups were sacrificed on the 27th day. The mean tumor volumes on the 27th day after treatment initiation were significantly decreased in curcumin and cisplatin combination group compared with controls (88.0 vs. 138.4 mm^3^; *p* < 0.05; Figure 11A and 11B). The mean tumor volumes of the curcumin or cisplatin alone groups were not statistically different from result of the control group (*p* = 0.353 and *p* = 0.275, respectively). There was no statistical difference in body weight among the four groups (Figure 11C). Therefore, curcumin and cisplatin combination treatment did not exhibit a significant decrease in body weight and was well tolerated.

## DISCUSSION

In the current study, we revealed that curcumin and cisplatin co-treatment enhanced apoptosis through ROS-mediated activation of ERK1/2 in human bladder cancer. We investigated the anticancer potential of curcumin for human bladder cancer control *in vitro and in vivo*. The results of several *in vitro* approaches suggested that, while either curcumin or cisplatin alone is fairly effective in suppressing proliferation and inducing apoptosis, co-treatment with the two agents is much more effective.

Wound healing and MTT assays demonstrated the antitumor effects of curcumin through reduced cancer cell migration and suppression of the proliferation of 253J-Bv and T24 cells. In addition, in line with the results of MTT assays, flow cytometry results after annexin V-FITC/PI staining showed significantly increased apoptotic cell death in the combined treatment group. In each cell line, combination therapy caused a significant increase in apoptosis, which was accompanied by upregulation of caspase 3 based on Western blot analysis and decreased cell viability based on the MTT assay. These findings indicate that curcumin at least partly exerts its synergistic antitumor activity with cisplatin in human bladder cancer through the induction of caspase-dependent apoptosis.

Although ROS are constantly generated during normal aerobic metabolism and xenobiotic exposure, excessive intracellular production of ROS generally leads to oxidative stress and causes damage to all components of the cell including proteins and lipids as well as DNA, ultimately inducing apoptosis [18, 19]. Recent advances have unambiguously revealed that curcumin is a intracellular pro-oxidant that generates ROS, causes oxidative stress, and subsequently induces apoptosis [20–22]. The mode of action of cisplatin has been shown to be associated with its ability to generate ROS as well as its ability to interact with DNA [7]. Combined treatments of some cell types with these two prooxidants are reported to have synergic effects on ROS generation. Our findings show that curcumin and cisplatin co-treatment exhibited a significant increase in ROS generation in both cell lines.

ERK is part of the MAPK superfamily, and is well known for its ability to modulate cell survival in response to external stimuli [23]. Several reports have found more complex roles for ERK in which the increase of ERK activity might promote apoptosis in specific environments. Protein kinase pathways such as the MAPK pathway are major oxidative stress-sensitive pathways in most cell types [24]. In particular, ERK is selectively activated in neuronal and renal epithelial cells upon exposure to oxidative stress and toxicants such as cisplatin, and inhibition of the ERK pathway blocks apoptosis [25].

Several studies have reported that curcumin potentiates ROS-dependent ERK activation and lethality in irradiated human cervical tumor cells [26], and that cisplatin-induced ERK activation is partly mediated through ROS generation [27]. In the current study, concomitant cisplatin and curcumin treatment significantly increased ERK phosphorylation in bladder cancer cells. These results seem to be consistent with several earlier studies, in which increased ERK activity was linked to the induction of cell death [28, 29]. Considering the crucial role of the ERK signal transduction axis as a cell death pathway, this finding indicates that curcumin and cisplatin combined treatment provides a more favorable milieu for apoptosis of bladder cancer through the activation of ERK signaling.

p53 protein, a transcription factor that acts as a tumor suppressor, seems to sense the extent of DNA damage and thereby determines whether to permit cell survival or activate the apoptotic program [7]. p21 protein expression is controlled by p53. The ERK pathway has been reported to cooperate to cause sustained cell cycle arrest requiring p21 protein expression [30]. The 253J-Bv cell line is p53 wild type whereas the T24 cell line is p53 mutant type [31, 32]. The exposure of 253J-Bv cells to concomitant treatment with cisplatin and curcumin increased p53 and p21 expression, indicating activation of p53 signaling by the co-treatment. However, p53 and p21 expression did not increase in T24 cells. The exposure of T24 cells to combination treatment suppressed STAT3 phosphorylation, suggesting suppression of STAT3 signaling by cisplatin and curcumin co-treatment. However, STAT3 phosphorylation was not suppressed in 253J-Bv cells. STAT3 overexpression is associated with a poor prognosis of bladder cancer [33]. Taken together, these results give rise to the tantalizing idea that the processes leading to drug-induced apoptosis differs according to p53 status. The present study is the first to suggest the potential of curcumin to activate different cisplatin-induced cell death pathways according to p53 status in human bladder cancer.

**Figure 12:**
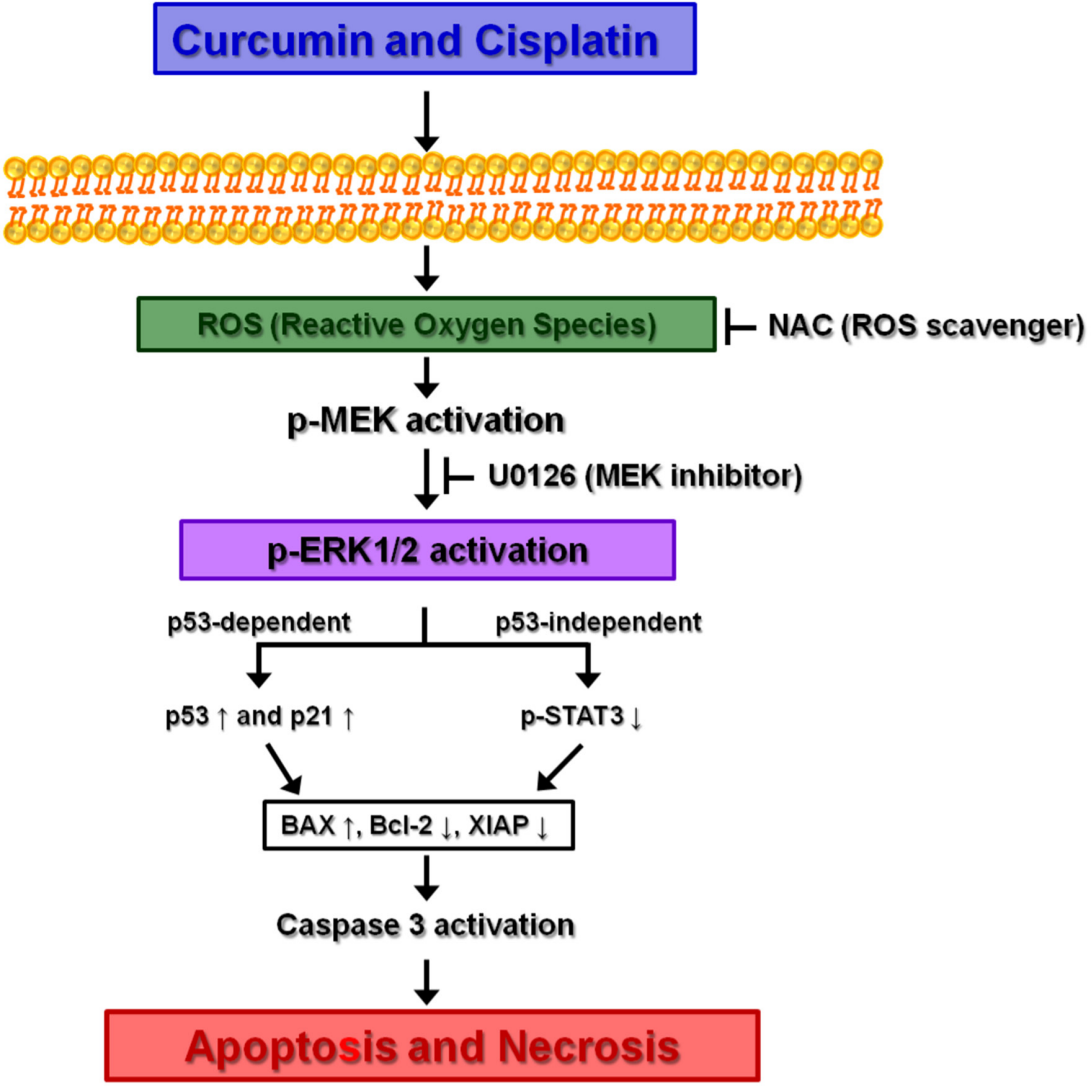
Proposed mechanism leading to apoptosis of bladder cancer cell lines via ROS-mediated activation of ERK1/2 with curcumin and cisplatin combination treatment

In summary, although the present study has the limitation of a small number of cancer cell lines tested, the data clearly reveal a synergistic interaction between curcumin and cisplatin in bladder cancer cells. Although curcumin alone showed only limited antitumor activity in bladder cancer cell lines, it synergistically potentiated cisplatin-induced apoptosis via ROS-mediated activation of ERK1/2. Also, our data indicate that the synergistic effect results in the targeting of different apoptotic pathways according to the p53 status of bladder cancer cells. Our proposed model for the molecular signaling pathway involved in this activity is summarized in Figure 12. Considering that human bladder cancer is most often diagnosed in the elderly, the ability to endure toxic treatment is low. Curcumin is safe and non-toxic for oral ingestion. Therefore, we believe curcumin and cisplatin co-treatment can be an effective and reliable approach for the management of human bladder cancer, although further comprehensive molecular pathway studies should be conducted to confirm the safety and antitumor activity for clinical application.

## MATERIALS AND METHODS

### Ethics statement

All experiments were approved and reviewed by the Institutional Animal Care and Use Committee of Samsung Biomedical Research Institute.

### Chemicals and reagents

Curcumin and cisplatin were purchased from Selleckchem (Houston, TX). Z-VAD-FMK (a pan-caspase inhibitor) and U0126 (an ERK inhibitor) were purchased from Calbiochem (San Diego, CA, USA). NAC (a ROS scavenger) was purchased from Sigma Chemical Company (St. Louis, MO, USA). Fluorescein isothiocyanate (FITC)-labeled annexin V and propidium iodide (PI) were purchased from BD Biosciences Pharmingen (San Diego, CA, USA). Dichlorodihydrofluorescein diacetate (H2DCFDA) was purchased from Molecular Probes (Waltham, MA, USA). Rabbit polyclonal antibodies against caspase-3, Bcl-2, p-MEK, MEK, p-ERK1/2, and ERK were purchased from Cell Signaling Technology (Beverly, MA, USA). Mouse polyclonal antibodies against p53 and p21 were purchased from Santa Cruz Biotechnology (Dallas, TX, USA). Mouse monoclonal antibody against XIAP was purchased from BD Biosciences Pharmingen (San Diego, CA, USA).

### Cell lines and culture conditions

Human bladder cancer cell lines 253J-Bv and T24 were purchased from the American Type Culture Collection (ATCC). 253J-Bv and T24 cells were cultured in DMEM and McCoy's 5A (Gibco BRL, Waltham, MA, USA), respectively, supplemented with 10% fetal bovine serum (FBS), penicillin (100 U/ml), and streptomycin (100 mg/ml) at 37°C under a humidified atmosphere with 5% CO_2._

### Pretreatment of 253J-Bv and T24 cells with various inhibitors

253J-Bv and T24 cells were pretreated with Z-VAD-FMK (10, 20, or 50 μM), NAC (1, 5, or 10 mM), or U0126 (5, 10, or 20 μM) for 1 h at 37°C. After preincubation, 253J-Bv and T24 cells were once washed with culture medium prior to treatment with curcumin and cisplatin. A lack of cytotoxicity was confirmed for all inhibitors at the tested concentrations.

### Cytotoxicity assay

Exponentially growing cells were plated in 96-well plates with 5×10^3^ cells/well and grown overnight in medium containing 10% FBS, penicillin (100 U/ml), and streptomycin (100 mg/ml) at 37°C under humidified atmosphere with 5% CO_2_. The medium was changed with fresh medium containing the designated drug conditions and the cells were incubated further for 24 and 48 h. Cell viability was determined using the Cell Counting Kit-8 (Dojindo Molecular Technologies Inc., Rockville, MD, USA) according to the technical manual provided with the kit.

### Annexin V-FITC/PI assay

Annexin V-FITC/PI staining was carried out by flow cytometry to detect apoptotic cells. Cells were seeded in 6-well plates at a concentration of 2×10^5^ cells/well and incubated overnight. The cells were then treated with different concentrations of curcumin, cisplatin, and combined curcumin and cisplatin for 24 and 48 h. After treatment, cells were collected, washed with PBS twice, and resuspended in the appropriate binding buffer. Cells were stained with annexin V-FITC (5 μl) and PI (10 mg/L), and incubated for 15 min in the dark at room temperature before analysis by flow cytometry (BD Biosciences Pharmingen, San Diego, CA, USA).

### Monolayer wound healing assay

For cell migration experiments, the cancer cells were seeded in a 24-well culture dish and incubated until 80% confluent. The monolayers were scratched with a 200-μl pipette tip. Cellular debris was then removed by washing using PBS, and the cancer cells were cultured in fresh medium. At different time points after scratching (0 and 24 h), images were obtained with an inverted microscope (Olympus, Tokyo, Japan) at 40× magnification. Cells were maintained at 37°C under a humidified atmosphere with 5% CO_2._

### Measurement of intracellular ROS production

Cells were incubated in 6-well plates (2×10^5^ cells/well) with the designated drugs for 6, 16, and 24 h to determine changes in ROS production. After incubation, the medium was aspirated off and intracellular ROS were detected using H2DCFDA. For fluorescence microscopy, H2DCFDA was added to the cancer cells in serum-free medium at a 10 μM final concentration and incubated at 37°C with 5% CO2 for 30 min. The medium with H2DCFDA was removed and the cancer cells were washed thrice with serum-free medium. Intracellular ROS were detected by fluorescence microscopy (Carl Zeiss Axiovert 200; Oberkochen, Germany). For flow cytometry, cells were obtained by centrifugation, washed two times with PBS, and subsequently resuspended in 500 μl H2DCFDA (10 μM) and incubated for 30 min in the dark at 37°C. Cells were immediately analyzed by flow cytometry at the end of incubation.

### Western blot assay

Cells, treated with or without designated drugs, were lysed with RIPA buffer (EMD Millipore Corporation, Billerica, MA, USA) and placed on ice for 20 min. After centrifugation at 12,000 X g for 5 min, the supernatants were carefully harvested and the protein concentration of each cell lysate was quantified using the Bradford assay. Samples containing 30 μg total protein were resolved in 10-15% SDS-PAGE gels, transferred to Immobilon-P polyvinylidene fluoride membranes (GE Healthcare Life Sciences, Pittsburgh, PA, USA) by electroblotting, and probed with appropriate primary antibodies at 4°C overnight. The membranes were subsequently soaked with horseradish peroxidase (HRP)-conjugated anti-rabbit or anti-mouse antibodies for 1 h at room temperature. Immunoreactive bands were detected using LumiGLO (Cell Signaling Technology, Beverly, MA, USA).

### *In vivo* studies

All animals received humane care according to the Guide for the Care and Use of Laboratory Animals prepared by the Institute of Laboratory Animal Resources (National Institutes of Health). Additionally, all animal procedures were performed in accordance with the Animal Experiment Guidelines of Samsung Biomedical Research Institute. For the subcutaneous delivery model, 6-week-old female BALB/c nude mice were obtained from Orient Bio (Korea). The effects of curcumin, cisplatin, and combination treatment on the tumorigenic potential of bladder cancer cells were analyzed by subcutaneous injection of 253J-Bv cells [5×10^6^ in 150 μL Hank's Balanced Salt Solution (HBSS) with matrigel (BD Biosciences Pharmingen, San Diego, CA, USA)] after administration of anesthesia. When mean tumor volume reached 32 mm^3^, 40 nude mice were randomly classified into four treatment groups: vehicle (as a control), curcumin, cisplatin, and combination curcumin and cisplatin treatment (n=10 per group). Group 1 mice were treated with cisplatin, which was administered once a week by intraperitoneal injection (5 mg/kg). Group 2 mice were treated with curcumin diluted in DMSO, which was administered 3 times per week by oral gavage (500 mg/kg). Group 3 mice received a combination treatment with the same concentrations of cisplatin and curcumin as in groups 1 and 2. The remaining 10 mice in the control group were administered sterile saline with the same timing and dosing schedule as that used for the treatment groups. The tumor volumes and body weights of mice were measured throughout the study. Tumor volume was estimated according to the formula: (longest diameter) × (shortest diameter)^2^/2. Mice were sacrificed using a standard method on the 27th day. The volumes of tumor and body weights of mice were compared among the groups.

### Statistical analysis

Results are presented as the mean ± SEM of at least three independent experiments. Statistical analyses were done using Student's *t* tests. Values with *p* <0.01 or 0.05 were considered statistically significant.
